# Morphological and Cytotoxic Effects of Metal Salts on Osteoblast Cells in Three‐Dimensional Spheroidal Cultures

**DOI:** 10.1155/jt/1047932

**Published:** 2026-09-26

**Authors:** Misael Vargas-López, José Luis Castrejón-Flores, Ángel Ernesto Bañuelos-Hernández, Fernando Gómez-Chávez, Ricardo García-Ruiz, Ma. Lourdes Rojas-Morales, Elizabeth Pérez-Hernández, Nury Pérez-Hernández

**Affiliations:** ^1^ Department of Bioprocesses, Interdisciplinary Professional Unit of Biotechnology, National Polytechnic Institute, Mexico City 07340, Mexico, ipn.mx; ^2^ Laboratory of Osteoarticular Diseases, National School of Medicine and Homeopathy, National Polytechnic Institute, Mexico City 07320, Mexico, ipn.mx; ^3^ Doctoral Program in Biological Sciences, National Autonomous University of Mexico, Graduate Unit, Mexico City 04510, Mexico, unam.mx; ^4^ National Laboratories of Experimental Services, Center for Research and Advanced Studies, National Polytechnic Institute, Mexico City 07360, Mexico, cinvestav.mx

**Keywords:** 3D culture, cell morphology, metal salts cytotoxicity, osteoblast

## Abstract

The skeleton provides structural support, organ protection, and mineral storage, making the long‐term biocompatibility of orthopedic implants essential. Although stainless steel and cobalt‐based alloys are widely used in these devices, corrosion can release metal ions that induce inflammatory and cytotoxic responses in surrounding bone tissue. Because most in vitro studies rely on conventional two‐dimensional (2D) cultures, three‐dimensional (3D) spheroids offer a more physiologically relevant model for evaluating metal ion toxicity. In this study, human fetal osteoblast (hFOB) spheroids were exposed to CrCl_3_, CoCl_2_, or NiCl_2_ at 100 or 500 μM for 12 days. Morphological alterations were evaluated by scanning electron microscopy and histology, while cytotoxicity and apoptosis were assessed using resazurin, live/dead fluorescence, and annexin V/propidium iodide staining. CrCl_3_ caused minimal effects at 100 μM and only moderate cytotoxicity at 500 μM. In contrast, CoCl_2_ and especially NiCl_2_ significantly reduced spheroid growth, induced retraction of cytoplasmic extensions, membrane damage, and increased apoptosis, with the most severe effects observed at 500 μM. Fluorescence and Annexin V staining confirmed a higher abundance of apoptotic bodies after CoCl_2_ and NiCl_2_ exposure than after CrCl_3_ treatment. These findings demonstrate that 3D osteo‐spheroids provide a physiologically relevant platform for investigating the biological effects of metal ions released from orthopedic implants and reveal that nickel and cobalt ions exert substantially greater cytotoxicity than chromium under prolonged exposure conditions.

## 1. Introduction

The skeleton is a versatile organ that helps in mobility, protects internal organs, and stores minerals. However, if bones are broken or lost because of trauma or illness, they need to be restored or replaced. To achieve this, bone plates, screws, or prostheses constructed from safe materials are required [1]. Metals are the primary category of biomaterials utilized in joint replacements and trauma interventions, due to their corrosion resistance, biocompatibility, mechanical strength, and fracture toughness [2].

Despite its susceptibility to corrosion compared to other metallic biomaterials, stainless steel remains widely used as a material for biomedical devices because of its low cost and workability [3]. It is composed of an iron (Fe)‐based alloy containing around 50% Fe by mass and over 12%–13% chromium (Cr), along with other components such as nickel (Ni), molybdenum (Mo), copper (Cu), titanium (Ti), niobium (Nb), and nitrogen (N) [4]. These alloying elements enhance its heat resistance, mechanical strength, and formability [5]. On the other hand, cobalt (Co)‐based alloy biomaterials comprise another critical group of metallic biomaterials that offer unique properties complementing iron Fe‐based alloys and are commonly used in cardiovascular, orthopedic, and dental applications [6].

Although metal medical devices are a reliable therapy for improving skeletal health outcomes, they are prone to wear and corrosion (tribocorrosion) from constant exposure to body fluids, tissues, and mechanical movements at implant interfaces [7]. Corrosion products, such as metal ions, can be toxic around the joint and, in some cases, lead to implant rejection. Experimental and ex vivo studies demonstrate that the biological effects of Cr^3+^, Co^2+^, and Ni^2+^ ions are strongly concentration‐dependent, with low to intermediate exposures (≈10–100 μM in many in vitro systems) predominantly induce inflammatory signaling, whereas higher local concentrations (≈500 μM or high ppm ranges) promote oxidative stress, cytotoxicity, and tissue necrosis [8–10]. As the concentration of metallic ions increases, they may infiltrate systemic and lymphatic circulation, and subsequently reach vital organs and inducing systemic toxicity [11]. The impact of systemic toxicity varies from mild to severe depending on the concentration of circulating metal levels, while local periprosthetic concentrations that exceed systemic values are considered the main drivers of severe tissue damage and implant failure [12, 13]. Some essential metal ions are also toxic even within their physiological ranges, complicating the interpretation of implant‐associated metal toxicity and its relationship with systemic disease [14].

Thus, tribocorrosion research in the medical field involves experiments designed to simulate in vivo wear and corrosion processes, aiming to understand the mechanisms of toxicity of biomaterials [15]. Two‐dimensional (2D) cultures of bone cells [9, 16] and immunological cells [10] have demonstrated that exposure to metal ions inhibits the transcription of bone‐regulating mediators, affecting mineralization processes, stimulating osteolytic activity, and increasing pro‐inflammatory responses in the implant area. These inflammatory responses involve the induction of cytokines and chemokines such as IL‐6, IL‐8, CCL2/MCP‐1, TNF‐α, and IL‐1β, linking metal ion exposure to immune cell recruitment, osteoimmune dysregulation, and early processes associated with implant rejection and osteolysis [8–10, 17]. Although these studies have significantly contributed to understanding the mechanisms of metal ion toxicity, 2D models have limitations in replicating physiological conditions, as these involve complex processes that comprise multiple cell types and signals.

Recent advances in tridimensional (3D) cell culture methods have expanded the use of in vitro assays that better mimic real tissue microenvironments. Cells cultured under these conditions exhibit *in vivo*–like responses due to their conformation, unlike those grown on 2D platforms [18]. From a molecular perspective, 3D systems reproduce diffusion gradients and sustained intercellular signaling that modulate oxidative stress, inflammasome activation, and cytokine production; experimentally, these models capture macrophage–fibroblast cooperation, matrix remodeling, and pro‐fibrotic or osteolytic responses that better resemble periprosthetic tissue behavior than 2D cultures [17, 19, 20].

Considering that the toxicity of metal ions has only been evaluated in 2D bone cell cultures and many mechanisms remain poorly understood, this work assessed the effects on cytotoxicity and cell morphological features of the metal salts chromium(III) chloride (CrCl_3_), cobalt(II) chloride (CoCl_2_), and nickel(II) chloride (NiCl_2_) on long‐term osteoblast spheroid 3D cultures (osteospheroids).

## 2. Materials and Methods

### 2.1. Cell Culture

Osteoblast cell line hFOB1.19 (CRL‐3602, ATCC) was cultured in Dulbecco’s modified Eagle medium F‐12 (DMEM F‐12, 30‐2006, ATCC 30‐2006) supplemented with 10% fetal bovine serum (FBS, S1560‐500 Biowest) and 1% 100 units/mL penicillin‐streptomycin (15240‐062 GIBCO) in a humidified incubator at 37°C and 5% CO_2_. The cells were cultured in 75‐cm^2^ culture flasks, and the medium was replaced two to three times per week.

### 2.2. Osteo‐Spheroid Formation

Osteo‐spheroids were generated using the liquid‐overlay technique [21]. Briefly, human fetal osteoblast (hFOB) cells, previously cultured as a 2D monolayer in 75‐cm^2^ culture flasks, were detached and seeded into 96‐well U‐bottom plates coated with a thin layer of 1% ultrapure agarose (16500‐100, Invitrogen) at a density of 10,000 cells per well. Culture medium was added to a final volume of 150 μL per well, and the cells were incubated for 72 h at 37°C in a humidified atmosphere containing 5% CO_2_ to allow spheroid formation.

Stock solutions (10 mM) of chromium(III) chloride (CrCl_3_) (450790, Sigma‐Aldrich), cobalt(II) chloride (CoCl_2_) (232696, Sigma‐Aldrich), and nickel(II) chloride (NiCl_2_) (339350, Sigma‐Aldrich) were prepared in ultrapure water. Working solutions of 100 and 500 μM were prepared by dilution in culture medium and sterilized by filtration through a 0.22‐μm membrane. Fresh working solutions were prepared every 3 days throughout the treatment period.

Following spheroid formation, cultures were exposed to 100 or 500 μM CrCl_3_, CoCl_2_, or NiCl_2_ for 12 days. The culture medium was replaced every 2–3 days by exchanging two‐thirds of the total volume with fresh medium containing the corresponding metal ion concentration. Spheroid integrity and diameter were monitored by phase‐contrast microscopy (Nikon Eclipse TS100) throughout the experimental period.

### 2.3. Treatment of Osteoblasts and Osteo‐Spheroids With Metal Ions and Cytotoxicity Assays

hFOB cells cultured as 2D monolayers (10,000 cells/well) and osteo‐spheroids were exposed to CrCl_3_, CoCl_2_, and NiCl_2_ at the previously described concentrations (100 and 500 μM) for 12 days. In both models, the culture medium was replaced every 3 days with fresh medium containing the corresponding metal‐ion solutions. Untreated cells and osteo‐spheroids cultured under identical conditions without metal salt exposure served as the respective control groups. All cultures were maintained at 37°C in a humidified atmosphere containing 5% CO_2_.

Cell viability was assessed using the resazurin reduction assay [22] on days 3, 6, 9, and 12 of treatment. Prior to the addition of the resazurin solution, cells and spheroids were gently washed with PBS to remove residual culture medium and extracellular metal ions. Monolayer cultures were incubated with 150 μL of a 30‐μg/mL resazurin solution (R7017‐5G, Sigma‐Aldrich) prepared in PBS for 4 h, whereas osteo‐spheroids were incubated under the same conditions for 24 h. Following incubation, 100 μL of supernatant from each well was transferred to a 96‐well flat‐bottom plate, and absorbance was measured at 570 nm using 600 nm as the reference wavelength with an Epoch Microplate Spectrophotometer (BioTek Instruments, Inc., Winooski, VT, USA). Cell viability was expressed as a percentage relative to the corresponding untreated control.

### 2.4. Live/Dead Fluorescence Staining of Spheroids

PBS was used to wash the spheroids three times before staining. The osteo‐spheroids were carefully transferred to sterile polypropylene microcentrifuge tubes and stained in suspension without prior fixation. They were then treated with a Live/Dead staining solution prepared in serum‐free culture medium containing 80‐μg/mL fluorescein diacetate (FDA) (D6883, Sigma‐Aldrich) and 200‐μg/mL propidium iodide (PI) (421301, BioLegend). Samples were incubated in the dark at 37°C with 5% CO_2_ for 30 min. Following incubation, the staining solution was removed, and the spheroids were washed three times with fresh culture medium, allowing a 10‐min interval between washes. Viable cells were labeled with FDA (Ex/Em: 494/521 nm, green fluorescence), whereas nonviable cells were labeled with PI (Ex/Em: 535/617 nm, red fluorescence). Immediately after staining, the intact spheroids were transferred onto glass microscope slides and imaged without fixation using a Nikon Eclipse 80i epifluorescence microscope equipped with a B‐2A filter set (Nikon 96302) for FDA and a G‐2A filter set (Nikon 96312) for PI. Images were captured under the corresponding fluorescence channels.

### 2.5. Histological Analysis

The osteo‐spheroids were collected after 3, 6, 9, and 12 days of metal salt treatment and individually placed into 0.65‐mL polypropylene microcentrifuge tubes (Costar, Cat. No. 3208, Corning Inc., Corning, NY, USA). They were fixed with 4% paraformaldehyde (P6148‐500G, Sigma‐Aldrich), embedded in paraffin (Paraplast, Leica 39601006), sectioned at a thickness of 2 μm, mounted onto microscope slides, and stained with hematoxylin (115938, Sigma‐Aldrich, St. Louis, MO, USA) and eosin (104134, Sigma‐Aldrich, St. Louis, MO, USA) (H&E stain). Additionally, Masson’s Trichrome Stain Kit (HT15, Sigma‐Aldrich, St. Louis, MO, USA) was used to evaluate extracellular collagen. Stained sections were examined under a light microscope (Zeiss Primostar 3, Germany), and images were captured using the Zeiss Axiocam 208 color camera.

### 2.6. Ultrastructural Analysis

For scanning microscopy analysis, the osteo‐spheroids were removed from the culture medium and washed three times in PBS. Subsequently, they were fixed in a 2.5% glutaraldehyde solution (16220‐Electron Microscope Sciences) for 1 h at room temperature. PBS was used to wash the samples three times for 5 min each, and then they were fixed with 1% osmium tetroxide (19192, Electron Microscope Sciences) in PBS for an hour at room temperature. After three 5‐min rinses in PBS, the spheroids gradually dehydrated with alcohol at 50%, 60%, 70%, 80%, and 90% concentrations, followed by three changes at 100% for 10 min each. Spheroids were dried at critical point (SAMDRI‐795, Tousimis), mounted, and coated with gold (20 nm thick) (Denton Vacuum, Desk V). Finally, the samples were analyzed using a scanning electron microscope (JEOL JSM‐6510LV, 8 kV).

Raw .tif extension files were processed with MATLAB 2025. Images at X2500 magnification, 886 × 1280 pixels in size, were enhanced using a CLAHE square filter with a contextual region of 2 × 2 pixels and a contrast‐enhanced limit of 0.001 (on a scale from 0 to 1, where a lower value corresponds to lower contrast) [23, 24]. The resultant images covered a rectangular region measuring 51.2 microns in width by 35.44 microns in height.

Extracellular vesicles (EVs) at the pixel level were manually labeled. Segmented regions were extracted for morphometric measures, including area, perimeter, equivalent diameter, significant axis length, minor axis length, maximum Feret diameter, minimum Feret diameter, eccentricity, circularity, extent, and length [25, 26]. The conversion of pixel measurements to microns was made at a ratio of 25 pixels per micron. Structural analysis of segmented regions was assessed with three texture indicators: entropy values in the range from 0 to 1 at maximum entropy [27]; local standard deviation (SD), measured with a disk‐shaped structural element with 2‐pixel radius [28]; and local range, measured with a disk‐shaped structural element with 2‐pixel radius [29]. The cumulative values for entropy, local SD, and local range were calculated by double numerical integration of their respective values in the *x* direction, followed by integration of these values in the *y* direction using the trapezoidal method [30]. The values for squared micron of pixel intensity, entropy, local SD, and local range were computed by the formulas: cumulative pixel intensity/area of the body; cumulative entropy/area of the body; cumulative local SD/area of the body; cumulative range/area of the body.

### 2.7. Annexin V‐FITC/PI Apoptosis Staining

Cellular spheroids were washed twice with PBS and incubated with Annexin V‐FITC and PI staining solution prepared in 1× binding buffer using an Annexin V‐FITC Apoptosis Detection Kit (Sigma‐Aldrich, St. Louis, MO, USA). Spheroids were stained with 5‐μL Annexin V‐FITC and 10‐μL PI per sample for 10 min at room temperature in the dark, following the manufacturer’s protocol. After incubation, samples were gently washed with binding buffer and immediately analyzed using a Nikon Eclipse 80i (Nikon Instruments Inc., Tokyo, Japan) equipped with a B‐2A filter set (Nikon 96302) for Annexin V‐FITC fluorescence and a G‐2A filter set (Nikon 96312) for PI. Annexin V‐FITC fluorescence identified apoptotic cells (Ex/Em: 488/519 nm, green), whereas PI staining identified late apoptotic or necrotic cells (Ex/Em: 535/617 nm, red). Samples were imaged immediately after staining to minimize signal loss.

### 2.8. Data Analysis and Statistics

For quantitative analyses, *n* = 10 independent spheroids were evaluated per experimental condition, unless otherwise indicated. Data are presented as mean ± SD. This applies to osteo‐spheroid diameter measurements, cell viability assays in 2D cultures and osteo‐spheroids, and EV quantification. Statistical analyses were performed using GraphPad Prism version 9.0 (GraphPad, San Diego, CA, USA). Osteo‐spheroid diameter and cell viability data were analyzed using one‐way analysis of variance (ANOVA) followed by Tukey’s multiple‐comparison test. EV data were analyzed using the Kruskal–Wallis test. Differences were considered statistically significant at *p* < 0.05. Significance levels were indicated as ^∗^ *p* < 0.05, ^∗∗^ *p* < 0.01, ^∗∗∗^ *p* < 0.001, and ^∗∗^ *p* < 0.0001.

## 3. Results

### 3.1. Osteo‐Spheroid Growth Is Delayed by the Presence of Metal Ions

**FIGURE 1:**
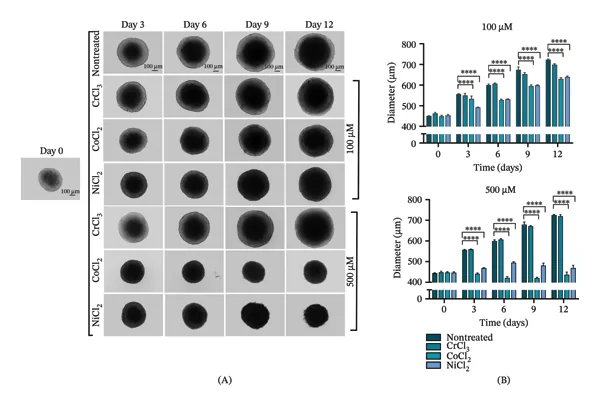
Effect of CrCl_3_, CoCl_2_, and NiCl_2_ on osteo‐spheroid morphology. (A) Macroscopic appearance of spheroids observed by phase‐contrast microscopy for 12 days following exposure to CrCl_3_, CoCl_2_, and NiCl_2_ at concentrations of 100 and 500 μM. (B) Spheroid diameter measured at 3, 6, 9, and 12 days post‐seeding, exposed to 100 and 500 μM of CrCl_3_, CoCl_2_, and NiCl_2_. Statistical analysis was performed using one‐way ANOVA with Tukey’s multiple comparison test (*n* = 10). The control corresponds to untreated osteo‐spheroids. ^∗∗∗∗^ *p* < 0.0001 compared to the control.

3D osteoblast spheroids were generated using the liquid‐overlay technique by seeding 1 × 10^4^ hFOB cells into a 96‐well culture plate. After 72 h, the osteo‐spheroids reached an initial diameter of approximately 443.6 μm. The spheroids grew at a rate of 1.2 × (550.1 μm) by day 3, 1.13 × (600.4 μm) by day 6, 1.5 × (670.8 μm) by day 9, and 1.6 × (718.6 μm) by day 12. After 3 days of culture, the osteo‐spheroids were exposed to 100 and 500 μM concentrations of CrCl_3_, CoCl_2_, and NiCl_2_. The sample sizes were monitored over 12 days for both the untreated and ion‐metal‐treated groups, as shown in Figure 1A. The nontreated group exhibited a gradual increase in diameter, reaching 718.6 μm on day 12 while maintaining its spherical architecture (Figure 1A,B). A comparable trend was observed in spheroids treated with 100 and 500 μM CrCl_3_, indicating the relative safety of this metal at the evaluated concentrations. On the other hand, treatment with 100 μM CoCl_2_ and NiCl_2_ resulted in spheroids that were 11% smaller than those in the metal‐free group, with diameters of 636.8 μm and 641.6 μm, respectively, on day 12. At 500 μM, spheroid growth was stopped entirely, with final diameters of 439.8 μm for CoCl_2_ and 457.3 μm for NiCl_2_. Additionally, osteo‐spheroids treated with these higher concentrations exhibited surface irregularities and a significant increase in the dark necrotic zone, which extended across nearly the entire spheroid (Figure 1A).

**FIGURE 2:**
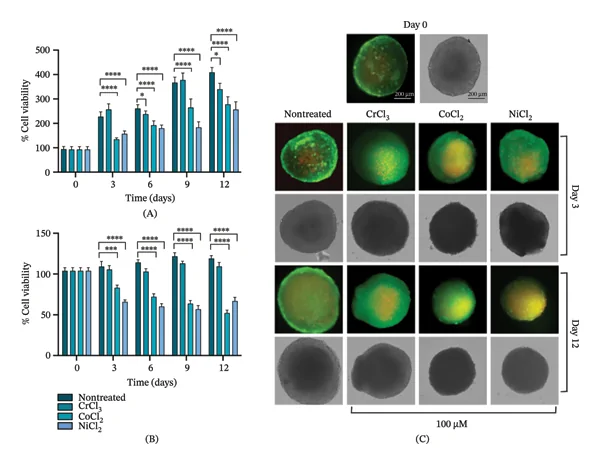
Effect of CrCl_3_, CoCl_2_, and NiCl_2_ at 100 μM on the viability of 2D and 3D cultures of hFOB1.19. (A) Cell viability determined by the resazurin assay in 2D cultures, or (B) in 3D osteo‐spheroids cultures of hFOB1.19 treated with CrCl_3_, CoCl_2_, and NiCl_2_ at 3, 6, 9, and 12 days at 100 μM. (C) Live/dead fluorescence staining of osteo‐spheroids after treatment with 100 μM CrCl_3_, CoCl_2_, and NiCl_2_ at days 3 and 12 post‐treatment. Green fluorescence indicates viable cells, while red fluorescence indicates dead cells. Representative bright‐field images of the corresponding spheroids are also shown. Scale bar = 200 μm. Data are expressed as mean ± SD (*n* = 10). Statistical analysis was performed using one‐way ANOVA followed by Tukey’s multiple comparison test. ^∗^ *p* < 0.05, ^∗∗∗^ *p* < 0.001, ^∗∗∗∗^ *p* < 0.0001 vs. nontreated control.

**FIGURE 3:**
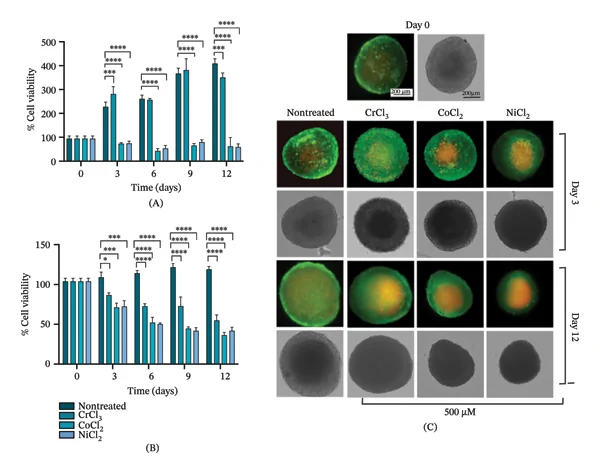
Effect of CrCl_3_, CoCl_2_, and NiCl_2_ at 500 μM on the viability of 2D and 3D cultures of hFOB1.19. (A) Cell viability determined by the resazurin assay in 2D cultures, or (B) in 3D osteo‐spheroids cultures of hFOB1.19 treated with CrCl_3_, CoCl_2_, and NiCl_2_ at 3, 6, 9, and 12 days at 500 μM. (C) Live/dead fluorescence staining of osteo‐spheroids after treatment with 500 μM CrCl_3_, CoCl_2_, and NiCl_2_ at Days 3 and 12 posttreatment. Green fluorescence indicates viable cells, while red fluorescence indicates dead cells. Representative bright‐field images of the corresponding spheroids are also shown. Scale bar = 200 μm. Data are expressed as mean ± SD (*n* = 10). Statistical analysis was performed using one‐way ANOVA followed by Tukey’s multiple comparison test. ^∗^ *p* < 0.05, ^∗∗∗^ *p* < 0.001, ^∗∗∗∗^ *p* < 0.0001 vs. nontreated control.

The cytotoxic effects of CrCl_3_, CoCl_2_, and NiCl_2_ on hFOB1.19 osteoblasts were assessed in 2D monolayer cultures and 3D osteo‐spheroids at metal salt concentrations of 100 μM (Figure 2) and 500 μM (Figure 3) over a 12‐day period using the resazurin metabolic activity assay.

At 100 μM, exposure to all three metal salts did not reduce cell viability in the 2D monolayer model compared with the nontreated control at 0 h (Figure 2A). On the contrary, elevations in metabolic activity were observed at several time points for CrCl_3_, CoCl_2_, and NiCl_2_, with values consistently at or above baseline throughout the 12‐day study period. This pattern suggests that sublethal metal‐ion exposure under monolayer conditions may stimulate rather than impair osteoblastic metabolic activity, possibly reflecting a hormetic or stress‐adaptive response facilitated by the unrestricted access to oxygen and nutrients inherent to 2D culture systems.

In contrast, the 3D osteo‐spheroid model revealed a markedly different, concentration‐dependent response to metal‐ion exposure at 100 μM (Figure 2B). CrCl_3_‐treated spheroids did not exhibit statistically significant reductions in cell viability relative to nontreated controls across the 12‐day observation period. However, CoCl_2_ induced a progressive and significant decline in viability, reaching a 24% reduction at day 3 (*p* < 0.0001) and a 56% reduction by day 12 (*p* < 0.0001). NiCl_2_ elicited a more pronounced cytotoxic effect, with a 40% decrease on day 3 and a 44% decrease on day 12, both statistically significant compared with the nontreated group (*p* < 0.0001). These findings establish a clear rank order of cytotoxicity in the 3D model at 100 μM: CrCl_3_ < CoCl_2_ < NiCl_2_.

Fluorescence Live/Dead staining of osteo‐spheroids treated with 100‐μM metal salts provided morphological corroboration of the viability data (Figures 2C and S1). At day 0, all spheroids displayed predominantly green fluorescence, consistent with a viable cell population distributed throughout the spheroid volume. By day 3, metal‐treated spheroids, particularly those exposed to CoCl_2_ and NiCl_2_, showed yellow‐orange fluorescence in their central regions, indicative of early necrotic core formation. By day 12, this central necrotic zone had expanded substantially in CoCl_2_‐ and NiCl_2_‐treated spheroids, evidenced by prominent orange‐red fluorescence cores surrounded by a residual viable green periphery. CrCl_3_‐treated spheroids showed comparatively modest changes in fluorescence pattern at this concentration. These observations demonstrate that metal‐ion‐induced cytotoxicity in 3D osteo‐spheroids follows a centripetal progression, with cell death initiating and intensifying within the hypoxic and nutrient‐deprived core over time.

When the concentration was increased to 500 μM, a striking and dose‐dependent divergence between the two culture models was observed. In 2D monolayer cultures (Figure 3A), metal salt treatment not only failed to reduce viability but also produced a marked and progressive supraphysiological increase in resazurin reduction for CrCl_3_ relative to baseline by day 12 (*p* < 0.0001 vs. nontreated). This paradoxical hyperactivation of metabolic activity in 2D cultures at 500 μM CrCl_3_, which substantially exceeded values observed at 100 μM, warrants careful interpretation, as it may reflect mitochondrial stress‐induced reductive metabolism, or compensatory upregulation of cellular activity independent of proliferative status. However, CoCl_2_ and NiCl_2_ at 500 μM showed a statistically significant reduction in cell viability.

By contrast, at 500 μM, the 3D osteo‐spheroid model exhibited significant and progressive cytotoxicity across all three metal compounds, starting on day 3 (Figure 3B). CrCl_3_ induced a 20% reduction in viability at day 3, which increased to 54% by day 12 (*p* < 0.0001). CoCl_2_ produced a 35% decline at day 3 and a 69% decline at day 12 (*p* < 0.0001). NiCl_2_ displayed the greatest toxicity overall, with viability decreasing by 34% on day 3 and by 65% by day 12 (*p* < 0.0001). These results confirm the cytotoxicity hierarchy established at 100 μM and demonstrate that the rank order CrCl_3_ < CoCl_2_ < NiCl_2_ is preserved and amplified at the higher concentration.

Live/Dead fluorescence imaging of spheroids at 500 μM further substantiated the quantitative viability findings (Figure 3C). On day 3, all metal‐treated spheroids exhibited large yellow‐orange cores with a reduced yet still present green periphery, indicating extensive early necrosis, substantially greater than that observed at 100 μM. By day 12, the necrotic core had expanded to encompass a significantly larger proportion of the spheroid volume in all treatment groups, with CoCl_2_‐ and NiCl_2_‐treated spheroids displaying the most prominent red fluorescence signal and the thinnest residual viable rims. Bright‐field images confirmed progressive structural alterations in spheroid morphology with increasing metal‐ion exposure and time, including loss of spheroid compactness and boundary definition at later time points. Collectively, the Live/Dead staining data at both concentrations were fully consistent with the resazurin‐based viability measurements, validating the 3D osteo‐spheroid as a sensitive model for detecting metal‐ion‐induced cytotoxicity in a microenvironment that more faithfully recapitulates the oxygen and nutrient gradients present in vivo.

Live/Dead fluorescence imaging also revealed a spatially organized pattern of cytotoxicity within the spheroids. Cell death progressed in a centripetal manner, with early formation of necrotic cores that expanded over time and with increasing metal ion concentration, while a thin viable peripheral layer remained. This gradient‐like distribution was consistently observed across treatment conditions and time points, and was particularly evident in CoCl_2_‐ and NiCl_2_‐treated spheroids, which exhibited the most extensive red fluorescence and the thinnest viable rims at day 12 (Figure 3C).

The discordance between 2D and 3D viability profiles across both concentrations underscores the critical influence of culture dimensionality on the assessment of metal ion cytotoxicity. The well‐nourished, homogeneous environment of monolayer cultures may mask or even invert the toxic effects observed at higher concentrations, whereas the diffusion‐limited and spatially heterogeneous architecture of the 3D spheroid makes it considerably more sensitive to metal‐induced cellular stress. As previously reported, the resazurin assay in 3D spheroids preferentially detects metabolically active cells at the spheroid periphery, which may account for the relatively sustained viability readings even at advanced time points despite the substantial necrotic cores visualized by Live/Dead staining [31].

Notably, the similar viability observed at day 0 and day 12 can also be attributed to cellular metabolic adaptation within the spheroid microenvironment. Cells in 3D spheroids adapt metabolically and establish nutrient and oxygen diffusion gradients that support long‐term viability, while the viability assay predominantly reflects metabolically active cells at the spheroid periphery, which remains well nourished even at later time points.

### 3.2. Morphological Response of Osteoblast Spheroids to Metal Ion Exposure

**FIGURE 4:**
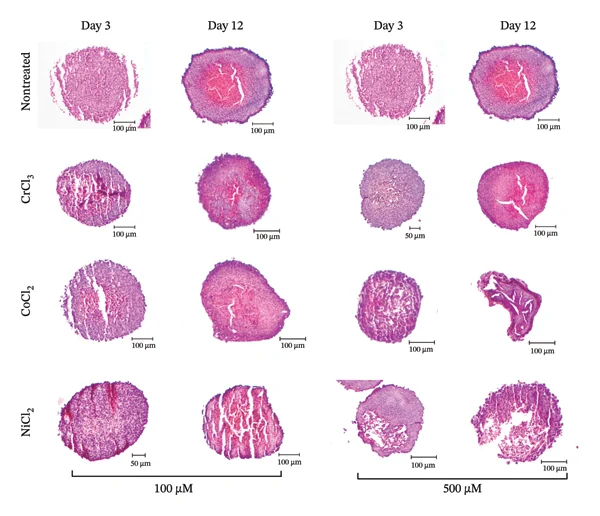
Hematoxylin and eosin (H&E)–stained osteo‐spheroids treated with metal ions and analyzed at Days 3 and 12 posttreatment. Spheroids were exposed to CrCl_3_, CoCl_2_, and NiCl_2_ at 100 and 500 μM. Representative images are shown.

To evaluate cell organization and morphology, photomicrographs of H&E‐stained osteo‐spheroid sections were obtained. After 3 days of seeding, morphological evaluation revealed that the nontreated spheroids consisted of compact aggregates of osteoblasts organized into three distinct zones: a peripheral proliferative zone, an intermediate quiescent zone, and a central necrotic zone. The surface osteoblasts exhibited rounded nuclei with finely dispersed chromatin, evident nucleoli, and a moderate amount of homogeneous, acidophilic cytoplasm. The intermediate zone contained rounded to elongated cells with smaller, hyperchromatic nuclei. In the central region of the spheroid, signs of karyorrhexis, pyknosis, and cellular debris were observed (Figure 4).

Osteo‐spheroids exposed to both concentrations of Cr^3+^ maintain their organization and size, like the nontreated group, until day 6. However, in the following days, karyorrhexis, pyknosis, and cellular debris became evident, particularly in the intermediate zone. Notably, a few osteoblasts exhibited nucleomegaly and chromatin arranged in coarse clumps, displaying an anaplastic appearance by day 6 at low chromium ion concentrations (Figure 4 and S2).

In contrast, osteo‐spheroids treated with Co^2+^ and Ni^2+^ ions showed significant structural changes at days 3, 6, 9, and 12 post‐treatment. The necrotic zone expanded progressively, exhibiting indentations and distortions that extended to the surface layer, especially at the higher concentration of 500 μM. Additionally, nuclear polymorphism increased, accompanied by irregular chromatin (Figures 4 and S2).

**FIGURE 5:**
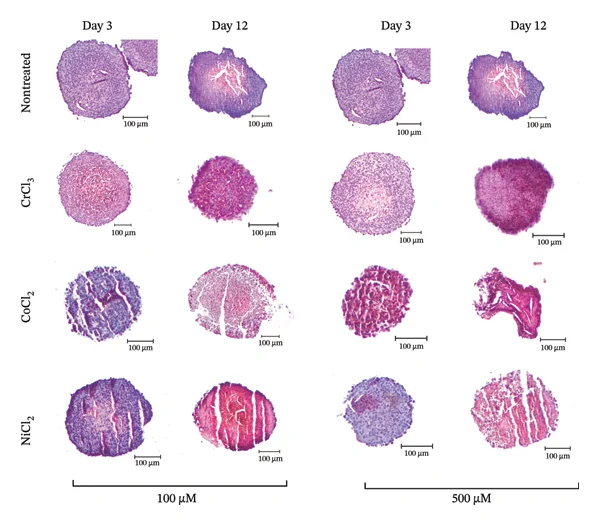
Masson’s trichrome staining osteo‐spheroids treated with metal ions and analyzed at days 3 and 12 post‐treatment. Spheroids were exposed to CrCl_3_, CoCl_2_, and NiCl_2_ at 100 and 500 μM. Representative images are shown.

In addition to H&E staining, Masson’s trichrome stain showed the presence of collagen in control spheroids at days 3, 6, and 9, with a diminution at day 12 (Figures 5 and S3). The collagen ratio decreases with Cr^3+^, Co^2+^, and Ni^2+^ treatment at 100 μM from day 9, while at 500 μM, the presence of collagen becomes scarcer from day 3, becoming more evident with trivalent chromium and divalent cobalt ions.

### 3.3. High‐Resolution Surface Morphological Analysis of Osteoblast Spheroids Exposed to Metal Ions

A detailed analysis of cell surface alterations in osteo‐spheroids following metal ion exposure was performed using scanning electron microscopy (SEM).

**FIGURE 6:**
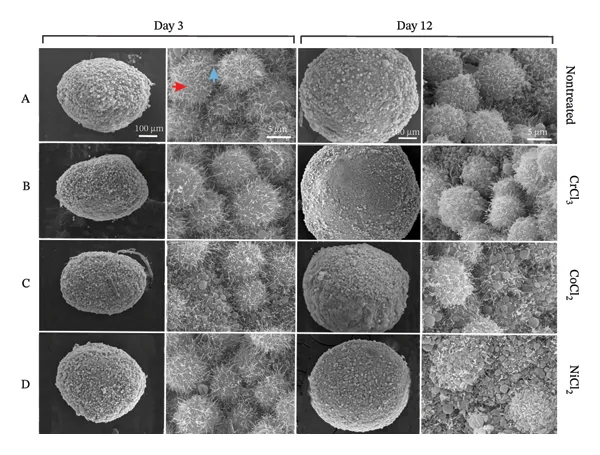
Electron micrographs of osteo‐spheroids subjected to CrCl_3_, CoCl_2_, and NiCl_2_ at a concentration of 100 μM. Photographs were taken on days 3 and 12 of the culture, both with and without CrCl_3_, CoCl_2_, or NiCl_2_. Low‐magnification images (100 μm) show the general shape of spheroids, while high‐magnification images (5 μm) show details of the cell surface. Since day 3, filopodia (red arrow) and cell contacts (blue arrow) have been visible.

**FIGURE 7:**
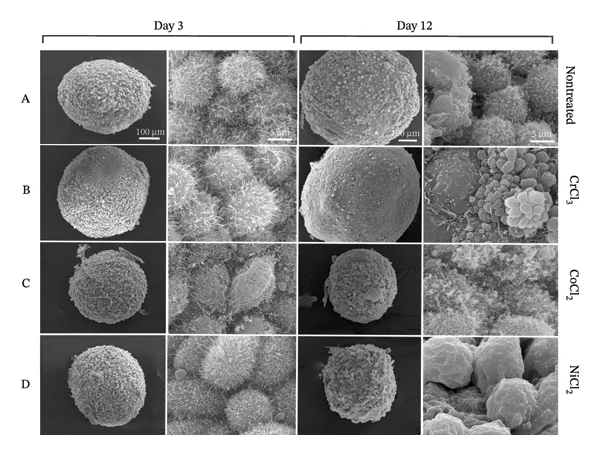
Electron micrographs of osteo‐spheroids subjected to CrCl_3_, CoCl_2_, and NiCl_2_ at a concentration of 500 μM. Photographs were taken on days 3 and 12 of the culture, both with and without CrCl_3_, CoCl_2_, or NiCl_2_. Low‐magnification images (100 μm) show the general shape of spheroids, while high‐magnification images (5 μm) show details of the cell surface.

At low magnification (100 μm), nontreated osteo‐spheroids appeared as densely packed, spherical cellular aggregates (Figures S4A, S5A, 6A, and 7A). In contrast, spheroids exposed to CoCl_2_ and NiCl_2_ (Figures 6C,D and 7C,D) exhibited a noticeable size reduction in a time‐ and concentration‐dependent manner, consistent with the diameter decrease described in Figure 1. In nontreated spheroids, osteoblasts appeared rounded and firmly adhered to one another. They exhibited a complex 3D network of membrane protrusions, including microvilli, filopodia, and elongated extensions, indicative of direct contact between neighboring cells (Figure 6A).

Cells of spheroids exposed to 100 or 500 μM CrCl_3_ displayed filopodia and cell–cell connections with no apparent alterations compared to nontreated controls (Figures 6B, 7B, S4B, and S5B). In contrast, CoCl_2_ treatment caused a pronounced retraction of cytoplasmic extensions in osteoblasts, particularly at the higher concentration (500 μM). The cells acquired an elongated and extended shape, with more expansive intercellular spaces, reduced cohesion, and the presence of reticulated material suggestive of cellular fragmentation and plasma membrane detachment, indicating irreversible damage (Figures 6C, 7C, S4C, and S5C).

When cells were treated with high levels of Ni^2+^, the number of membrane filopodia and microvilli was significantly reduced, reflecting diminished cellular contact and adhesion capacity. The cells lost their round shape and exhibited grooves, cracks, and disorganized areas on their surfaces, which are typical of cellular stress. However, there was no reticulated material, such as Co^2+^. Additionally, a reduction in the fibrillar extracellular matrix was noted, and by day 12, osteoblasts exhibited swelling with a completely smooth surface, signifying significant cytotoxic effects (Figures 6D, 7D, S4D, and S5D).

**FIGURE 8:**
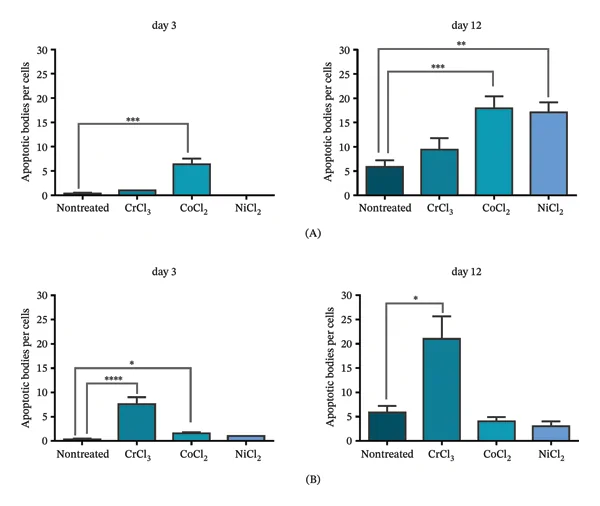
Number of extracellular vesicles per ten cells in osteo‐spheroids treated with CrCl_3_, CoCl_2_, and NiCl_2_ at 100 μM (A) and 500 μM (B) on days 3 and 12. Statistical analysis was performed using multiple‐comparison Kruskal–Wallis tests (*n* = 6). ^∗^ *p* < 0.05, ^∗∗∗∗^ *p* < 0.0001 compared to the nontreated group.

EVs of different sizes were evident on the osteoblast membrane. These were manually labeled at the pixel level for analysis, and the segmented regions were used to measure morphometric properties like area, perimeter, equivalent diameter, major axis length, minor axis length, maximum Feret diameter, minimum Feret diameter, eccentricity, circularity, extent, and length. See Table S1 and Figure S6 for details. Using these criteria, a selection of EVs was made based on the following characteristics: the diameter was greater than 1 μm to exclude smaller subtypes, such as exosomes; the area was greater than 20; the perimeter was greater than 3; and the corresponding entropy was greater than 0.75. Although eccentricity was not used as a selection parameter, most of the analyzed structures exhibited eccentricity values below 0.5, indicating that they are predominantly spherical in shape. These EVs are likely apoptotic bodies (ABs), although the presence of other structures, such as autophagosomes, cannot be excluded [32, 33]. After selecting the EVs in the osteo‐spheroids for each condition, a count was conducted for every 10 clearly defined cells. The graphs for days 3 and 12 are illustrated in Figure 8.

In the case of CrCl_3_, the number of vesicles remained relatively low at 100 μM, but a marked increase was observed at 500 μM after 12 days of exposure. Together with the viability tests, this result suggests that higher concentrations of Cr^3+^ induce a stronger apoptotic response. For CoCl_2_, a similar pattern was observed at 100 μM; however, at 500 μM, extensive cell destruction was evident, resulting in the absence of EVs. This indicates the activation of a cell death mechanism other than apoptosis, which may account for the overall viability results shown in Figures 2 and 3. Likewise, NiCl_2_ treatment prevented the presence of EVs and produced outcomes comparable to those of cobalt ions. However, exposure appeared to trigger a distinct cellular appearance, characterized by the loss of spherical morphology and the emergence of a very flat cell surface.

**FIGURE 9:**
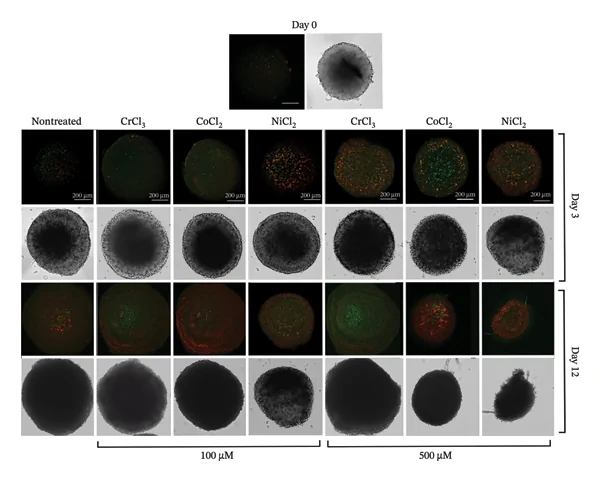
Annexin V/propidium iodide (PI) staining of osteo‐spheroids treated with CrCl_3_, CoCl_2_, and NiCl_2_, at 100 and 500 μM of each metal for 3 and 12 days. Green fluorescence (Annexin V) indicates early apoptotic cells, while red fluorescence (PI) corresponds to late apoptotic or necrotic cells. Representative images are shown.

These quantitative observations are supported by Annexin V/PI staining (Figure 9 and S7). At early time points, an increase in Annexin V‐positive cells was detected, indicating early apoptosis. In contrast, at later stages, a predominance of PI‐positive cells was observed, reflecting loss of membrane integrity and progression toward late apoptosis and secondary necrosis.

At 100 μM, CoCl_2_ and NiCl_2_ treatments led to a higher number of ABs and increased fluorescence intensity compared to CrCl_3_, indicating a stronger pro‐apoptotic effect. At 500 μM, spheroids treated with CoCl_2_ and NiCl_2_ exhibited a higher proportion of PI‐positive cells and greater structural disintegration than those treated with CrCl_3_, supporting a shift toward necrotic cell death under these conditions.

Overall, these results demonstrate that metal exposure induces a progressive transition from early apoptosis to late apoptosis and necrosis in a time‐ and dose‐dependent manner, with CoCl_2_ and NiCl_2_ exerting a more pronounced cytotoxic effect than CrCl_3_.

## 4. Discussion

Aseptic loosening, local osteolysis, and cardiovascular complications are among the well‐documented toxicological consequences of metal ion release from orthopedic implants [34–36]. Despite advances in alloy technology, the cellular effects of individual metal ions released from commonly used biomaterials remain incompletely understood, particularly in physiologically relevant 3D models. In the present study, osteo‐spheroids were exposed to CrCl_3_, CoCl_2_, and NiCl_2_, which dissociate in aqueous culture medium to release the corresponding metal cations (Cr^3+^, Co^2+^, and Ni^2+^) and chloride ions. Accordingly, the Results section refers to the administered metal salts, whereas the Discussion focuses on the biological effects associated primarily with the released metal cations, which are generally considered the main bioactive species responsible for the observed cellular responses. In this context, the present study provides a characterization of the effects of Cr^3+^, Co^2+^, and Ni^2+^ on hFOB spheroids.

In CoCr orthopedic implants, metal ion release occurs through wear and corrosion processes, resulting in simultaneous exposure to multiple ionic species rather than isolated ions. Cobalt and chromium ions are frequently detected in periprosthetic fluids, with chromium typically present at low micromolar concentrations. Importantly, previous studies have shown that combined Co^2+^/Cr^3+^ exposure can induce enhanced biological effects compared to single‐ion treatments, suggesting additive or synergistic interactions [37, 38].

While the present study was designed to characterize the individual effects of Co^2+^, Cr^3+^, and Ni^2+^ in a controlled 3D osteoblast spheroid model, this reductionist approach provides a necessary baseline for interpreting more complex exposure scenarios. A clear understanding of single‐ion toxicity is essential for the rigorous evaluation of interaction effects under co‐exposure conditions.

Future studies should therefore implement factorial co‐exposure designs using physiologically relevant concentrations to systematically assess potential synergistic or antagonistic interactions between metal ions.

Our research showed that osteo‐spheroids derived from hFOB cells, without any differentiation stimuli, maintained their spherical shape and increased in size over time, reaching 718.6 μm after 12 days. This is 1.6 times larger than their original diameter. Interestingly, Cr^3+^ did not change the size or shape of the spheroids, but at high levels (500 μM), it did kill cells. The literature indicates that trivalent chromium ions are typically regarded as safe for osteoblast‐like cells, such as MG63 and SAOS‐2 [16], as well as for human primary osteoblasts [9], at concentrations below 500 μM in 2D culture. Our findings extend these observations by defining cytotoxic thresholds in a 3D culture system that more closely mimics the in vivo microenvironment.

Under the experimental conditions used in this study (DMEM/F‐12, pH ∼7.4, 37°C, 10% FBS, 5% CO_2_), chromium was introduced exclusively as Cr^3+^, and its oxidation to Cr^6+^ is expected to be both thermodynamically and kinetically unfavorable. The culture environment, enriched with serum components and intracellular reducing agents such as thiols and ascorbate, provides a predominantly reducing milieu that stabilizes Cr^3+^ and disfavors its oxidation. Accordingly, the biological effects observed in this study can be attributed to the trivalent chromium species, which are representative of the corrosion products released from stainless steel and CoCr alloys under physiological conditions [39].

The cytotoxic effect of Cr^3+^ identified in this study appears to be primarily associated with apoptotic processes, particularly when EVs are interpreted as ABs. However, previous studies have shown that metals such as iron and lead can also induce autophagy [40, 41], suggesting that additional cell death mechanisms may be involved in response to chromium exposure. The concurrent presence of ABs and putative autophagic structures, as observed by SEM, supports the activation of multiple cell death pathways in osteoblasts following Cr^3+^ treatment, thereby amplifying the overall cytotoxic response in osteo‐spheroids.

At the cellular level, Cr^3+^ exerts its effects primarily through its high affinity for negatively charged biomolecules, forming stable coordination complexes with DNA phosphate groups, nuclear proteins, and membrane lipids, thereby compromising DNA integrity and triggering p53‐dependent apoptotic cascades [42]. Although Cr^3+^ is considered far less genotoxic because it has lower cell membrane permeability than its hexavalent counterpart, evidence suggests that it might interfere with chromatin remodeling and induce oxidative DNA lesions under sustained exposure conditions [43]. Importantly, whole‐genome transcriptomic analysis of primary human osteoblasts exposed to cobalt and chromium ions has revealed concentration‐dependent activation of stress‐response pathways, including upregulation of genes involved in oxidative stress, cell cycle arrest, and matrix metalloproteinase activity [43]. Interestingly, it has been reported that Cr^3+^ does not impair osteoblast function and the expression of various members of the TGF‐beta signaling cascade in osteoblast‐like cells, which is consistent with our findings [44]. Overall, Cr^3+^‐treated cell viability remained similar to that of the control group, but electron microscopy revealed more ABs in the spheroids. This apparent discrepancy can be attributed to the limitations of metabolically based assays, which primarily reflect overall cellular activity and may not detect early apoptotic events, during which cells can preserve metabolic function. These findings were substantiated by Annexin V/PI staining, which revealed an elevation in Annexin V‐positive cells, thereby confirming the induction of apoptosis. These findings collectively suggest that Cr^3+^ exposure facilitates apoptotic processes that are not promptly detected by standard viability assays, especially in 3D spheroid models.

The treatment with Co^2+^, on the other hand, reduced spheroid size in a concentration‐ and time‐dependent manner, with growth inhibition evident even at low concentrations. Osteoblast death was further evidenced by karyorrhexis and pyknosis, which extended into the intermediate zone of the osteo‐spheroids, along with a reduction in the number of putative ABs. These findings are consistent with previous reports describing the cytotoxic effects of divalent cobalt ions [45], primarily through apoptosis, in MG‐63 osteoblast‐like cells [46, 47] and in primary human osteoblast cultures under monolayer conditions [48].

This effect has been associated with well‐documented mechanisms, including excessive generation of reactive oxygen species (ROS), which promotes lipid peroxidation and protein oxidation, ultimately leading to mitochondrial and cytoskeletal damage, chromatin condensation, and chromosomal aberrations [49].

The cytotoxic and antiproliferative effects of Co^2+^ observed in our osteo‐spheroids are consistent with its well‐established role as an inhibitor of prolyl hydroxylase domain proteins (PHDs), the oxygen‐sensing enzymes that target hypoxia‐inducible factor‐1α (HIF‐1α) for proteasomal degradation under normoxic conditions. By competitively displacing iron from the catalytic site of PHDs, Co^2+^ stabilizes HIF‐1α independently of oxygen tension, inducing a state of pseudohypoxia [50]. This transcriptional reprogramming activates downstream targets, including vascular endothelial growth factor (VEGF), glucose transporters, and glycolytic enzymes, profoundly altering osteoblast metabolism and function [51]. While low‐dose cobalt‐mediated HIF‐1α activation has been exploited as a pro‐angiogenic strategy in bone tissue engineering applications—particularly in cobalt‐doped bioactive glass scaffolds—the concentrations required for therapeutic angiogenesis are orders of magnitude below those tested in the present study. At the cytotoxic doses employed here (100–500 μM), Co^2+^ additionally activates NF‐κB and MAPK signaling cascades, promoting pro‐inflammatory cytokine production and mitochondrial membrane depolarization, ultimately converging on caspase‐dependent apoptosis [43, 44]. The apoptosis observed in live/dead staining and the ABs measured by SEM in osteo‐spheroids treated with CoCl_2_ in the present study are consistent with this mitochondria‐to‐nucleus apoptotic cascade, extending from the spheroid intermediate zones, consistent with progressive ion penetration.

In addition, osteoblasts within the spheroids exposed to Co^2+^ exhibited a reduced number of filopodia, consistent with cytoskeletal damage and the probable oxidation of actin filaments involved in their formation. This observation aligns with previous reports, which show that osteoblast‐like Saos‐2 cells exposed to cobalt nanoparticles exhibit a more rounded morphology, accompanied by a reduction in tubular actin filaments [52, 53].

Ni^2+^ was the most cytotoxic ion compared to Cr^3+^ and Co^2+^, a trend corroborated by prior studies in 2D cultures, where the predominant distribution of osteoblasts in the Sub G1 population of the cell cycle indicated apoptosis as the primary mechanism of cell death [54, 55].

Additionally, the morphological changes observed in osteo‐spheroids exposed to Ni^2+^ were different from those caused by Co^2+^ [21]. In addition to cells exhibiting diminished contacts due to the loss of filopodia and a significant reduction in EVs, the presence of a markedly damaged plasma membrane, characterized by extensive grooves and cracks, may indicate cellular necrosis. Previous research utilizing osteoblast‐like cells and primary osteoblasts in monolayers has documented analogous alterations, whereby exposure to Ni ions or Ni^2+^ nanoparticles induced cell rounding, reduced cell size, and diminished the expression of cytoskeletal proteins, including actin [46, 56, 57].

The superior cytotoxicity of Ni^2+^ relative to Cr^3+^ and Co^2+^ observed in this study aligns with its multitarget molecular profile. Like cobalt, nickel ions can inhibit PHDs and stabilize HIF‐1*α* by displacing iron, thereby inducing pseudohypoxia‐associated transcriptional changes [50]. However, Ni^2+^ exerts additional epigenetic effects by inhibiting histone demethylases, particularly jumonji‐domain‐containing enzymes that require iron and 2‐oxoglutarate as cofactors, leading to global histone hypermethylation and transcriptional silencing of tumor suppressor genes. Furthermore, Ni^2+^ is a potent activator of the NF‐κB signaling axis, as well as the MAPK, IRF3, and NLRP3 inflammasome pathways, which collectively amplify pro‐inflammatory and pro‐apoptotic responses [58]. In the context of bone biology, these converging mechanisms simultaneously impair osteoblast differentiation. It may reduce collagen synthesis, disrupt cytoskeletal organization, and, as observed in this study, promote cell death, thereby explaining the extensive plasma membrane damage and filopodial loss observed in Ni^2+^‐treated spheroids. From a regenerative medicine perspective, these findings underscore the need for strict control of nickel ion release from nickel‐containing stainless‐steel alloys and nickel‐titanium (nitinol) devices in bone‐adjacent environments [59].

While this study did not explicitly assess the mechanisms governing extracellular matrix regulation, the reduction in collagen fibers observed through Masson’s trichrome staining in osteo‐spheroids treated with Cr^3+^, Co^2+^, and Ni^2+^ indicates that matrix production is compromised even at minimal ion concentrations [16]. This finding corroborates earlier studies demonstrating that Cr^6+^ significantly inhibits extracellular collagen synthesis at concentrations as low as 0.5 mM, whereas Cr^3+^ has a less pronounced yet still detectable impact, especially in 2D culture systems. Our results corroborate these observations in 3D models, reinforcing the hypothesis that extended exposure to Cr^3+^ may adversely affect collagen synthesis in a microenvironment that more accurately reflects in vivo conditions. Additionally, the downregulation of collagen‐related genes reported in the literature aligns with the substantial decrease in total collagen observed during CoCl_2_ and NiCl_2_ treatment [16, 44]. These findings suggest that metal ions affect osteoblast viability and may also disrupt extracellular matrix homeostasis, necessitating further investigation through targeted studies on collagen synthesis and remodeling pathways.

A critical aspect of interpreting cytotoxicity data in 3D spheroid models is the spatial distribution of metal ions within the spheroid. Unlike monolayer cultures, where all cells are uniformly exposed, 3D spheroids establish radial gradients of oxygen, nutrients, and metabolic waste products as a function of diffusion distance from the periphery [60].

In agreement with this framework, spatial organization within the spheroids revealed a clear gradient‐dependent pattern of cytotoxicity. Live/Dead staining demonstrated a centripetal progression of cell death, characterized by early formation of a necrotic core that progressively expanded over time and with increasing metal ion concentration, while a thin viable peripheral layer was preserved. These findings are directly visualized in the Live/Dead fluorescence panels (Figures 2C and 3C), providing experimental evidence of the “outer layers versus inner core” gradient characteristic of 3D cellular systems.

These gradients create three functionally distinct zones: an outer proliferating layer with full access to ions and nutrients, an intermediate quiescent zone with reduced metabolic activity, and a hypoxic or necrotic core with severely limited diffusion [60]. Metal ion penetration into spheroids should be governed by the balance between inward diffusion and cellular uptake and binding at the periphery, thereby creating a metal ion sink that limits penetration depth. Ions with high affinity for extracellular matrix proteins and membrane receptors, such as Ni^2+^, which binds avidly to histidine residues, may be partially sequestered at the spheroid surface, producing a steep concentration gradient toward the interior. This phenomenon may explain why viability losses detected by the resazurin assay at early time points (day 3) are disproportionately lower than the morphological damage observed by electron microscopy, as the metabolically active peripheral cells, which dominate the fluorescence signal, remain viable, whereas inner cells sustain progressive injury. As the spheroid grows and the necrotic core expands over time, the effective diffusion barrier diminishes, allowing deeper ion penetration and explaining the amplified cytotoxicity observed on day 12 across all treatment conditions. Future studies employing synchrotron X‐ray fluorescence microscopy or laser ablation inductively coupled plasma mass spectrometry (LA‐ICP‐MS) on spheroid cryosections would enable direct spatial mapping of metal ion distribution within osteo‐spheroids, providing mechanistic clarity on the relationship between ion penetration depth and the observed patterns of cell death, as has been reported in the deciphering of the mechanistic in drug delivery in 3D cultures, including A2780 human ovarian cancer cell spheroids and osteosarcoma spheroid models [61, 62].

The observed reduction in the number of EVs in metal‐treated osteo‐spheroids raises an important mechanistic question about the interplay between vesicular trafficking and intracellular metal‐ion homeostasis. Under physiological conditions, cells employ multiple mechanisms to prevent toxic metal accumulation, including metallothionein chelation, lysosomal sequestration, and active efflux via vesicular export pathways [42]. Lysosomes, in particular, function as acidic microreactors that concentrate and chemically transform metal species through their low‐pH, redox‐active environment [63]. The biogenesis of EV subtypes—including exosomes derived from multivesicular bodies (MVBs)—is intimately linked to the endolysosomal pathway, and metal ions that impair lysosomal membrane integrity or vesicular fusion events may simultaneously disrupt both degradative and secretory routes of metal detoxification [42, 63]. Consequently, impaired vesicular transport induced by Cr^3+^, Co^2+^, or Ni^2+^ exposure could create a self‐reinforcing cycle of intracellular metal accumulation: as EV biogenesis is compromised, the capacity for metal efflux decreases, driving further intracellular accumulation, lysosomal dysfunction, and ultimately mitochondrial damage and cell death. This mechanism is particularly plausible in the 3D spheroid context, where cells at intermediate depths may experience sublethal ion concentrations sufficient to impair vesicular transport without immediate membrane disruption, producing the progressive cytotoxicity pattern observed over the 12‐day study period. Direct validation of this hypothesis would require quantification of metal content in isolated EV fractions alongside intracellular metal mapping, representing a compelling avenue for future investigation.

## 5. Conclusion

This study demonstrates that hFOB‐derived osteo‐spheroids exhibit distinct responses following exposure to CrCl_3_, CoCl_2_, and NiCl_2_, which dissociate in culture medium to release Cr^3+^, Co^2+^, and Ni^2+^ ions. The released metal cations were associated with different patterns of cell death and alterations in cellular morphology, suggesting that the toxicity profiles of these metal species may contribute differently to the biological effects of alloy degradation products around joint implants. While CrCl_3_ (as a source of Cr^3+^) exhibited cytotoxicity only at high concentrations, CoCl_2_ impaired spheroid growth and altered cytoskeletal organization. NiCl_2_ produced the greatest cytotoxicity, causing pronounced morphological alterations. These findings highlight the value of 3D culture models for elucidating cell death pathways and extracellular matrix modifications that are not fully captured in conventional 2D systems. Overall, these data provide relevant insights into the cellular responses induced by metal ions released from biomedical alloys and underscore the need for safer biomaterial design and further investigation into their long‐term effects on bone tissue homeostasis.

## Author Contributions

Misael Vargas‐López: writing–review and editing, methodology, and investigation. José Luis Castrejón‐Flores: writing–review and editing and supervision. Ángel Ernesto Bañuelos‐Hernández: supervision, validation, and methodology. Fernando Gómez‐Chávez: writing–review and editing, supervision, and formal analysis. Ricardo García‐Ruiz: writing–review and editing and methodology. Ma. Lourdes Rojas‐Morales: methodology and investigation. Elizabeth Pérez‐Hernández: writing–review and editing, validation, supervision, conceptualization, and formal analysis. Nury Pérez‐Hernández: writing–review and editing, writing–original draft, validation, supervision, methodology, funding acquisition, formal analysis, and conceptualization.

## Funding

This research was supported by Secretaría de Investigación y Posgrado, IPN, Project 20250062.

## Conflicts of Interest

The authors declare no conflicts of interest.

## Supporting Information

Additional supporting information can be found online in the Supporting Information section.

## Supporting information


**Supporting Information** Figure S1. Live/dead fluorescence staining of osteo‐spheroids after treatment with 100 and 500 μM CrCl_3_, CoCl_2_, and NiCl_2_ at Days 6 and 9 posttreatment. Figure S2. Hematoxylin and eosin (H&E)–stained osteo‐spheroids treated with metal ions and analyzed at Days 6 and 9 posttreatment. Figure S3. Masson’s trichrome staining osteo‐spheroids treated with metal ions and analyzed at Days 6 and 9 posttreatment. Figure S4. Electron micrographs of osteo‐spheroids treated with CrCl_3_, CoCl_2_, and NiCl_2_ at 100 μM at Days 6 and 9. Figure S5. Electron micrographs of osteo‐spheroids treated with CrCl_3_, CoCl_2_, and NiCl_2_ at 500 μM at Days 6 and 9. Figure S6. Electron micrograph of an osteo‐spheroid treated with 100 μM CoCl_2_ at Day 3 . Figure S7. Annexin V/PI staining of osteo‐spheroids treated with CrCl_3_, CoCl_2_, and NiCl_2_, at 100 μM and 500 μM of each metal for 6 and 9 days. Table S1. Morphometric properties of EVs from spheroids treated with 100 μM CoCl_2_ on day.

## Data Availability

The data that support the findings of this study are available from the corresponding author upon request.
