# Supplementary material for: Morphological and Cytotoxic Effects of Metal Salts on Osteoblast Cells in Three‐Dimensional Spheroidal Cultures
Source: J Toxicol. 2026 Sep 26;2026:1047932. doi: 10.1155/jt/1047932 (PMC13615670; doi:10.1155/jt/1047932)
Supplement: Supplementary file 1 [file JT-2026-1047932-s001.zip › Supporting information.docx]

**Morphological and Cytotoxic Effects of Metal Salts on Osteoblast Cells in Three-Dimensional Spheroidal Cultures**

**Supp****orting Information**

**Figure S1.** Live/Dead fluorescence staining of osteo-spheroids after treatment with 100 and 500 µM CrCl₃, CoCl₂, and NiCl₂ at days 6 and 9 post-treatment. Green fluorescence indicates viable cells, whereas red fluorescence indicates dead cells. Representative bright-field images of the corresponding spheroids are also shown. Scale bar = 200 µm.

**Figure S2.** Hematoxylin and eosin (H&E)-stained osteo-spheroids treated with metal ions and analyzed at days 6 and 9 post-treatment. Spheroids were exposed to CrCl₃, CoCl₂, and NiCl₂ at 100 µM and 500 µM. Representative images are shown.

**Figure S3. Masson’s trichrome staining osteo-spheroids treated with metal ions and analyzed at days 6 and 9 post-treatment.** Spheroids were exposed to CrCl₃, CoCl₂, and NiCl₂ at 100 µM and 500 µM. Representative images are shown.

**Figure S4.** **Electron micrographs of osteo-spheroids treated with CrCl₃, CoCl₂, and NiCl_2_ at 100 µM at days 6 and 9.** Low-magnification images (100 μm) show the general shape of spheroids, while high-magnification images (5 μm) show details of the cell surface.

**Figure S5.** **Electron micrographs of osteo-spheroids treated with CrCl₃, CoCl₂, and NiCl_2_ at 500 µM at days 6 and 9.** Low-magnification images (100 μm) show the general shape of spheroids, while high-magnification images (5 μm) show details of the cell surface.

**Figure S6. Electron micrograph of an osteo-spheroid treated with 100 µM CoCl₂ at day 3.** Left: high-magnification image (5 µm to 10 µm) showing labeled extracellular vesicles (EVs). Right: selected entropy objects of two EVs.

**Figure S7. Annexin V/propidium iodide (PI) staining of osteo-spheroids treated with CrCl₃, CoCl₂, and NiCl₂, at 100 µM and 500 µM of each metal for 6 and 9 days.** Green fluorescence (Annexin V) indicates early apoptotic cells, while red fluorescence (PI) corresponds to late apoptotic or necrotic cells. Representative images are shown.
